# Whole Genome Sequence of the Heterozygous Clinical Isolate *Candida krusei* 81-B-5

**DOI:** 10.1534/g3.117.043547

**Published:** 2017-07-07

**Authors:** Christina A. Cuomo, Terrance Shea, Bo Yang, Reeta Rao, Anja Forche

**Affiliations:** * Infectious Disease and Microbiome Program, and; †† Broad Institute of Massachusetts Institute of Technology and Harvard, Cambridge, Massachusetts 02142; † Worcester Polytechnic Institute, Biology and Biotechnology, Massachusetts 01609; ‡ Department of Biology, Bowdoin College, Brunswick, Maine 04011

**Keywords:** *Candida krusei*, 81-B-5, heterozygosity, LOH, mating type locus, transporters, Genome Report

## Abstract

*Candida krusei* is a diploid, heterozygous yeast that is an opportunistic fungal pathogen in immunocompromised patients. This species also is utilized for fermenting cocoa beans during chocolate production. One major concern in the clinical setting is the innate resistance of this species to the most commonly used antifungal drug fluconazole. Here, we report a high-quality genome sequence and assembly for the first clinical isolate of *C. krusei*, strain 81-B-5, into 11 scaffolds generated with PacBio sequencing technology. Gene annotation and comparative analysis revealed a unique profile of transporters that could play a role in drug resistance or adaptation to different environments. In addition, we show that, while 82% of the genome is highly heterozygous, a 2.0 Mb region of the largest scaffold has undergone loss of heterozygosity. This genome will serve as a reference for further genetic studies of this pathogen.

*Candida krusei* is a diploid, heterozygous yeast with an estimated chromosome number of six (Whelan and Kwon-Chung 1988; Samaranayake and Samaranayake 1994; Essayag *et al.* 1996; Jacobsen *et al.* 2007). *C. krusei* is an opportunistic fungal pathogen in immunocompromised patients, and, unlike other major pathogenic *Candida* species (*e.g.*, *C. albicans*), does not belong to the CUG clade (CTG is translated as serine rather than leucine) (Mühlhausen and Kollmar 2014). *Pichia kudriavzevii* (synomyn *Issatschenkia orientalis*) is the teleomorphic (sexual) state of *C. krusei* (Kurtzman *et al.* 1980); it is one of the main fermenters of cocoa beans important for the development of chocolate aroma (Jespersen *et al.* 2005; Nielsen *et al.* 2005; Pedersen *et al.* 2012), and is a potential producer of bioethanol and phytase (Chan *et al.* 2012).

In recent years, human fungal infections caused by *C. krusei* have increased in the clinic, due mainly to its innate resistance to the azole class of antifungal drugs, specifically to fluconazole (FLU) (Orozco *et al.* 1998; Guinea *et al.* 2006; Desnos-Ollivier *et al.* 2008; Lamping *et al.* 2009; Ricardo *et al.* 2014). FLU is a first line antifungal, also used as a prophylactic treatment in the intensive care unit, and breakthrough Candidemia is increasingly caused by non-*albicans* species including *C. krusei* (Lischewski *et al.* 1995; Chaudhary *et al.* 2015; Cuervo *et al.* 2016). Moreover, there are incidences of resistance to the echinocandin class of antifungals, which are the drug of choice to fight *C. krusei* infections (Forastiero *et al.* 2015). Therefore, identifying the exact mechanisms that underlie drug resistance, and in particular azole resistance, is of utmost importance.

The mechanisms causing *C. krusei* to be innately resistant to fluconazole are not well understood. Studies have shown that *C. krusei* Erg11p, the drug target, is significantly less susceptible to FLU inhibition than most other fungal Erg11p proteins (Orozco *et al.* 1998; Fukuoka *et al.* 2003), and that efflux pumps such as Abc1p are at least partially responsible for the innate FLU resistance of *C. krusei* (Lamping *et al.* 2009). Other studies have shown that overexpression of both *ERG11* and *ABC2* genes might be responsible for resistance to other azole drugs (He *et al.* 2015).

One approach to examine the basis of drug resistance of *C. krusei* is to mine the genome sequence for genes with potential roles in resistance such as novel drug pumps or transporters. To date, genome sequences have been generated for five environmental strains of *C. krusei* (*P. kudriavzevii*); the only available high quality assembly is of strain 129 isolated from fermented masau fruits (Van Rijswijck *et al.* 2017). A genome sequence for clinical isolates is still lacking. Here we report a high-quality genome sequence and assembly for clinical isolate *C. krusei* 81-B-5 (Scherer and Stevens 1987; Beckerman *et al.* 2001) into 11 scaffolds generated with PacBio sequencing technology. Gene annotation and comparative analysis revealed a unique profile of transporters that could play a role in drug resistance or adaptation to different environments. In addition, we show that while 82% of the genome is highly heterozygous, a 2.0 Mb region of the largest scaffold has undergone loss of heterozygosity.

## Materials and Methods

### Sequencing methods and preparation

High molecular weight genomic DNA was isolated from *C. krusei* strain 81-B-5 (Scherer and Stevens 1987; Beckerman *et al.* 2001) using a QIAGEN Genomic-tip 500/G kit (catalog # 10262). DNA was adapted using the SMRTbell Template Prep Kit, and sequenced using PacBio Technology (P6-C4 chemistry). A total of three SMRTcells were run, generating a total of 266,621 subreads with mean read length 5758, with a total of 1,535,304,314 bases (∼140× coverage). DNA was also adapted for Illumina sequencing, and a total of 16,953,446 paired 101b reads was generated on a HiSeq 2500.

### Assembly and annotation

An initial assembly was generated using HGAP (Chin *et al.* 2013) version 3 with smrtanalysis-2.3.0; HGAP was run with an estimated genome size of 14 Mb. As the genome was highly heterozygous, we also evaluated Falcon and Falcon-unzip (Chin *et al.* 2016) assemblies after Quiver polishing (using smrtanalysis-2.3.0). Falcon assembly settings were as follows: length_cutoff = 10,000; length_cutoff_pr = 500; pa_HPCdaligner_option = –v –dal4 –t16 –e.70 –11000 –s1000 –M32; ovlp_HPCdaligner_option = –v –dal4 –t32 –h60 –e.96 –1500 –s1000 –M32; pa_DBsplit_option = –x500 –s1000; ovlp_DBsplit_option = –x500 –s1000; falcon_sense_option = –output_multi–min_idt 0.70–min_cov 2–max_n_read 15–n_core 6; overlap_filtering_setting = –max_diff 72–max_cov 100–min_cov 2–bestn 12–n_core 24. Falcon-unzip was run with default settings other than specifying settings for the Sun Grid Engine (SGE) compute environment. Quiver (Chin *et al.* 2013) was then run on both assemblies to improve the consensus accuracy; initial evaluation of assemblies prior to Quiver polishing revealed a high rate of base errors. In both the HGAP and Falcon assemblies, contigs representing the alternative haplotype were identified based on high identity alignments to larger contigs in the assembly and roughly half the sequence depth in these regions; these alternative contigs were removed from both assemblies. Mitochondrial contigs were identified in all assemblies and set aside; the largest mitochondrial contig of 51.3 kb was assembled by HGAP assembly and smaller mitochondrial sequences were also identified in the Falcon or Falcon-unzip assemblies.

All assemblies were annotated to evaluate gene set completeness. An initial gene set was predicted using BRAKER (Hoff *et al.* 2016) to execute Genemark-ET with the parameter –fungus; tRNAs were predicted using tRNAscan (Lowe and Eddy 1997) and rRNAs predicted using RNAmmer (Lagesen *et al.* 2007). Genes containing PFAM domains found in repetitive elements or overlapping tRNA/rRNA features were removed. Genes were named and numbered sequentially.

### SNP calling

Illumina reads were aligned to the HGAP *C. krusei* genome assembly using the Burrows-Wheeler Aligner (BWA) 0.7.12 mem algorithm (Li 2013) with default parameters. Across the total of 16,306,945 aligned reads, the average depth was 140.0×. BAM files were sorted and indexed using Samtools (Li *et al.* 2009) version 1.2. Picard version 1.72 was used to identify duplicate reads and assign correct read groups to BAM files. BAM files were locally realigned around INDELs using GATK (McKenna *et al.* 2010) version 3.4-46 RealignerTargetCreator and IndelRealigner. SNPs and INDELs were called from all alignments using GATK version 3.4-46 HaplotypeCaller in GVCF mode with ploidy = 2, and genotypeGVCFs was used to predict variants in each isolate. Sites were filtered using variantFiltration with QD < 2.0, FS > 60.0, MQ < 40.0, and ReadPosRankSum < −8.0. Individual genotypes were then filtered if the minimum genotype quality <50, percent alternate allele <0.8, or depth <10.

### Repeat analysis

*De novo* repetitive elements were identified with RepeatModeler version open-1.0.7 (www.repeatmasker.org/RepeatModeler/); this identified only one unclassified element of length 1.3 kb and further analysis revealed that this region contains an Arg-tRNA. To identify copies of previously identified elements, RepeatMasker version 4.0.5 (www.repeatmasker.org) was used to identify copies of the RepBase22.04 fungal elements. *C. albicans* major repeat sequences were retrieved from the Candida Genome Database assembly version 22 (Skrzypek *et al.* 2017). Sequences were compared to the *C. krusei* assembly using BLAST; no similarity was found at 1e−5, requiring an alignment length of ≥100 bases.

### Comparative genomic analysis

Gene sets of *C. krusei*, *C. lusitaniae* (Butler *et al.* 2009), *C. albicans* (Jones *et al.* 2004; Van Het Hoog *et al.* 2007), *P. pastoris* (Love *et al.* 2016), *C. glabrata* (Dujon *et al.* 2004), and *S. cerevisiae* (Cherry, *et al.* 2012) were compared using BLASTP (e < 1e−10) and orthologs identified from the BLASTP hits using Orthomcl (Li *et al.* 2003). For the *CDR*/*MDR* gene family, protein sequences were aligned using MUSCLE (Edgar 2004) and alignments trimmed using TrimAl (Capella-Gutiérrez *et al.* 2009) with setting –gappyout. The best amino acid replacement model was selected using ProtTest version 3.4.2 (Darriba *et al.* 2011). A phylogeny was inferred using RAxML version 8.2.4 (Stamatakis 2014) with model GAMMALG and 1000 bootstrap replicates.

### Karyotype analysis

Chromosome plugs were prepared using the CHEF Genomic DNA plug kit (Bio-Rad) with the following modifications: single colonies were transferred to 5 ml YPD broth (1% yeast extract, 2% bacto peptone, and 2% glucose), and incubated at 30° for 18 hr in a roller incubator. The lyticase incubation step was done for 24 hr, and the CHEF plugs were incubated with Proteinase K for 48 hr. For the final washing steps, plugs were transferred to 5 ml tubes containing 3 ml of wash buffer. Chromosomes were separated in a 0.8% agarose gel (certified Megabase agarose (Bio-Rad), in 0.5× TBE buffer) with a DRII pulsed-field gel electrophoresis (PFGE) apparatus (Bio-Rad) using the following run parameters: Block1; 300 sec initial and final switch, 3.9 V/cm, at a 120° angle for 24 hr at 10°, Block 2; 1000 sec initial and final switch at 2.7 V/cm at a 120° angle for 48 hr at 10°. The gel was stained with ethidium bromide (0.5 µl/ml) for 15 min, destained in distilled water for 15 min and photographed. *S. cerevisiae* and *Hansenula wingei* chromosome size markers (Bio-Rad) were used for size estimates.

### Phenotypic analyses

Standard growth and media conditions have been previously described (Chauhan and Kruppa 2009). An Etest was used to determine the MIC for fluconazole (Pfaller *et al.* 2003). Briefly, overnight cultures were grown in YPD, washed and diluted to a final A600 of 0.1, then 500 μl were spread onto RPMI1640 agar plates (buffered with MOPS). After a 30 min preincubation, an Etest strip was applied and plates were incubated at 30° for 48 hr. The susceptibility endpoint reported was read at the first growth inhibition ellipse.

To confirm the nonfilamentous phenotype of *C. krusei*, 3 ml of YPD overnight cultures were washed, cells were counted, and 10^3^ cells were transferred to wells of a 12-well Petri plate containing 1 ml RPMI1640 with 10% fetal bovine serum. Plates were incubated at 37° and microscopic images were taken at 2, 4, and 8 hr. *C. albicans* (SC5314) and *S. cerevisiae* (S288c) were used for positive (filamenting) and negative (nonfilamenting) controls, respectively.

### Data availability

All genome sequence data (reads, assembly, and annotation) is available in GenBank under BioProject PRJNA381554. This Whole Genome Shotgun project has been deposited at DDBJ/ENA/GenBank under the accession NHMM00000000. The version described in this paper is version NHMM01000000.

## Results and Discussion

### Strain sequenced and phenotypic characterization

The sequenced isolate *C. krusei* 81-B-5 (number 653 in Scherer strain collection) was collected from a clinical source prior to 1987 (Scherer and Stevens 1987). To confirm that strain 81-B-5 is resistant to FLU, this isolate was grown in the presence of FLU and an Etest was done confirming the drug resistant phenotype with a minimum inhibitory concentration (MIC) of 32 µg/ml (Supplemental Material, Figure S1), which is considered highly resistant (Pfaller *et al.* 2003; Espinel-Ingroff *et al.* 2014). To verify the nonfilamentous phenotype of *C. krusei*, cells were exposed to serum, a potent inducer of filamentation and microscopically observed over time. Our results confirm that *C. krusei* does not filament as compared to *C. albicans* (Figure S2).

### Genome sequencing and assembly

We sequenced the genome of *C. krusei* using PacBio technology to generate long reads. Early attempts to assemble the genome using Illumina or 454 data had resulted in highly fragmented assemblies (Chan *et al.* 2012; JQFK00000000 and BBOI00000000), and we reasoned that the heterozygosity detected in multilocus sequence typing (MLST) analyses (Jacobsen *et al.* 2007) could likely complicate short read assembly. In assembling the genome, we compared assemblies generated using three methods, hierarchical genome assembly process (HGAP), Falcon, and Falcon-unzip, and evaluated metrics for the haploid consensus produced by HGAP and Falcon to the diploid assembly produced by Falcon-unzip. In addition to evaluating assembly metrics, we predicted gene calls on each assembly and evaluated gene set completeness as an additional metric.

While overall assembly statistics were similar, both assembly and gene metrics were superior on the HGAP version (Table S1). The HGAP assembly contained only 11 scaffolds, whereas nearly twice this number was generated by Falcon or in the Falcon-unzip primary contigs. The total assembly size in these assemblies was very similar, with 63 kb more sequence in the Falcon-unzip assembly compared to the HGAP assembly. As our prior experience in assembling diploid *Candida* genomes revealed that consensus errors can result in gene truncations where haplotypes are merged in a haploid assembly (Butler *et al.* 2009), we compared gene metrics across the three assemblies. Gene sets were compared to *C. albicans* to evaluate completeness. By this measure of gene content, the gene set on the HGAP assembly appears to be of higher quality, with 135 more *C. albicans* orthologs compared to the Falcon assembly, and 303 more than the Falcon-unzip. Gene length was also compared and not found to be very different; genes in the Falcon-unzip assembly were 16 bases larger on average than those in the HGAP. We also evaluated gene content on the second haplotype assembled by Falcon-unzip; these scaffolds totaled 2.1 Mb less than the other assemblies, and correspondingly fewer genes were predicted (Table S1). The completeness of the HGAP gene set was also evaluated by comparing to the BUSCO set of 1438 fungal orthologs (Simão *et al.* 2015). A total of 1278 appear complete in the *C. krusei* gene set. By comparison, this count is similar to the 1296 complete orthologs in *C. lusitaniae*, but fewer than the 1412 orthologs identified in the *C. albicans* genome, which has been extensively annotated (Braun *et al.* 2005; Butler *et al.* 2009; Bruno *et al.* 2010; Skrzypek *et al.* 2017). Based on considering both the assembly and gene metrics, we selected the HGAP assembly to represent the genome (Table 1). Compared to a previously reported draft genome (Chan *et al.* 2012), our assembly is more contiguous (11 contigs compared to 626 contigs for the PA12 assembly); the total size and gene number are comparable, with our assembly including 0.5 Mb more of sequence and a slightly higher gene count. A recently reported genome of isolate 129 using a hybrid of PacBio and Illumina in the assembly was also more fragmented (260 contigs) (Van Rijswijck *et al.* 2017); this assembly was larger in terms of total size (0.77 Mb), suggesting that some regions may be represented by both haplotypes in this assembly.

**Table 1:** *C. krusei* genome statistics

Scaffolds	11
Contigs	11
Total bases	10,910,993
Contig N50 length	1.36 Mb
Contig N90 length	543 kb
SNP rate	1 SNP/ 340 bases
GC content	38.42%
Repeat content	2.15%
Protein coding genes	4,949

**Figure 1:**
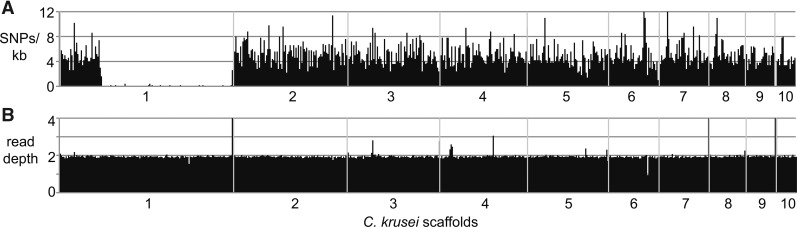
Genome-wide heterozygosity and genome coverage. (A) Heterozygous SNP positions are plotted across the assembly scaffolds in windows of 5 kb. (B) Normalized read depth is plotted across the assembly scaffolds in windows of 5 kb. Scaffold 11, consisting of around six ribosomal DNA repeats, is not depicted.

This *C. krusei* genome shows a high rate of heterozygous SNP variants, and one large region of loss of heterozygosity on scaffold 1. Using Illumina sequence, a total of 32,131 heterozygous SNPs was identified, for an average rate of 1 SNP every 340 positions, which is higher than rate reported in many *C. albicans* isolates (Butler *et al.* 2009, Hirakawa *et al.* 2015). While SNPs were distributed across the genome assembly, a 2.0 Mb region of scaffold 1 has undergone loss of heterozygosity; the first 0.6 Mb of scaffold 1 has a typical frequency of SNP variants; however, very few variants were detected across the remainder of the scaffold (Figure 1A). This homozygous region is not represented in the alternate haplotype contigs assembled by Falcon-unzip, and this difference explains the smaller assembly size of the Falcon-unzip alternate haplotype (Table S1). All of scaffold 1 is present at diploid levels, and we detect no large regions of aneuploidy in this isolate (Figure 1B).

The *C. krusei* genome contains very few repetitive sequences. A search for conserved repetitive elements classified only 0.40% of the assembly as interspersed repeats, with an additional 1.89% of sequence representing simple repeats. There are no regions with significant similarity (BLAST, 1e−5) to the *C. albicans* major repeat sequences (*Materials and Methods*). The average GC content is 38.4%, which is intermediate compared to related species such as *C. albicans* (33.5%) or *C. lusitaniae* (44.5%) (Jones *et al.* 2004; Van Het Hoog *et al.* 2007; Butler *et al.* 2009).

### Chromosome structure

**Figure 2:**
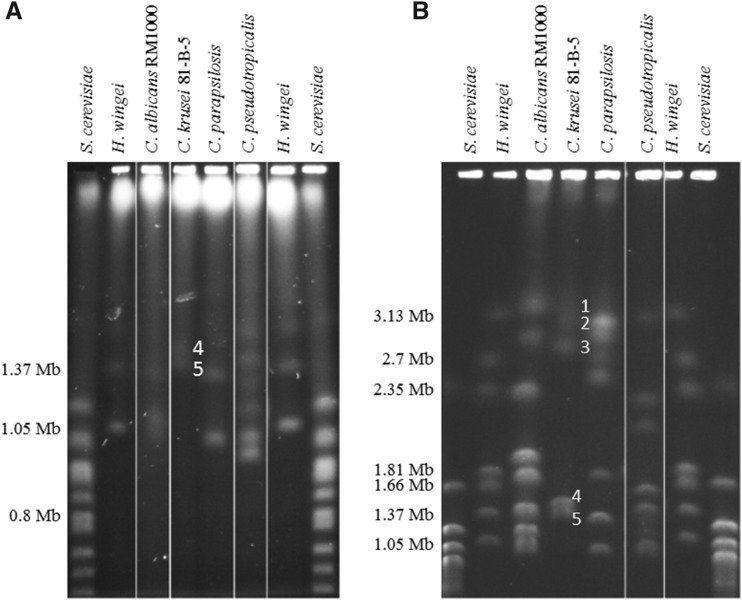
Karyotype analysis of *C. krusei* strain 81-B-5 reveals five chromosomal bands. (A) Short run to separate chromosomes smaller than 2 Mb. (B) Long run to separate all chromosomes. The chromosomes for *C. krusei* are labeled one through five. Several other *Candida* species were run as references; *S. cerevisiae* and *H. wingei* standards (Bio-Rad) were used for chromosome size estimation of *C. krusei* chromosomes.

PFGE was used previously to estimate the number of chromosomes for clinical and environmental isolates of *C. krusei* (Iwaguchi *et al.* 1990; Doi *et al.* 1992; Dassanayake *et al.* 2000; Jespersen *et al.* 2005). Based on the chromosomal patterns, it was estimated that *C. krusei* has a total of four to six chromosomes: around two to four large chromosomes (∼2.8–3.5 Mb), and two small chromosomes (∼1.4 Mb). PFGE for *C. krusei* strain 81-B-5 showed around five chromosomal bands, which were numbered based on size with one being the largest chromosome (Chr1) (Figure 2). Chromosome sizes were estimated based on the *H. wingei* and *S. cerevisiae* chromosome standards, and three non-*krusei Candida* species with known chromosome sizes (Doi *et al.* 1992; Butler *et al.* 2009): Chr1 (3.1 Mb), Chr2 (2.9 Mb), Chr3 (2.7 Mb), Chr4 (1.4 Mb), and Chr5 (1.3 Mb) (Figure 2). Based on these sizes the estimated genome size is 11.4 Mb, which is in good agreement with the size of the genome assembly. CHEF Southern analysis will be required to assign each scaffold to its appropriate chromosome, and additional work would be needed to establish the order and orientation of scaffolds along each chromosome.

By searching for tandem repeats at scaffold ends, we identified a candidate telomeric repeat (ATTGTAACACACCTCGCTCCTAGTTCAT). This repeat is found at five scaffold ends, including the start of scaffold 1, the end of scaffold 3, both ends of scaffold 4, and the start of scaffold 10. This suggests that scaffold 4 is a complete chromosome, and that four other scaffolds extend to the telomeres. rDNA repeats are detected at the end of scaffold 1, across scaffold 11, and the end of scaffold 9, suggesting that these scaffolds may be joined in a single chromosome to form a continuous rDNA array.

### Comparative genomics

**Figure 3:**
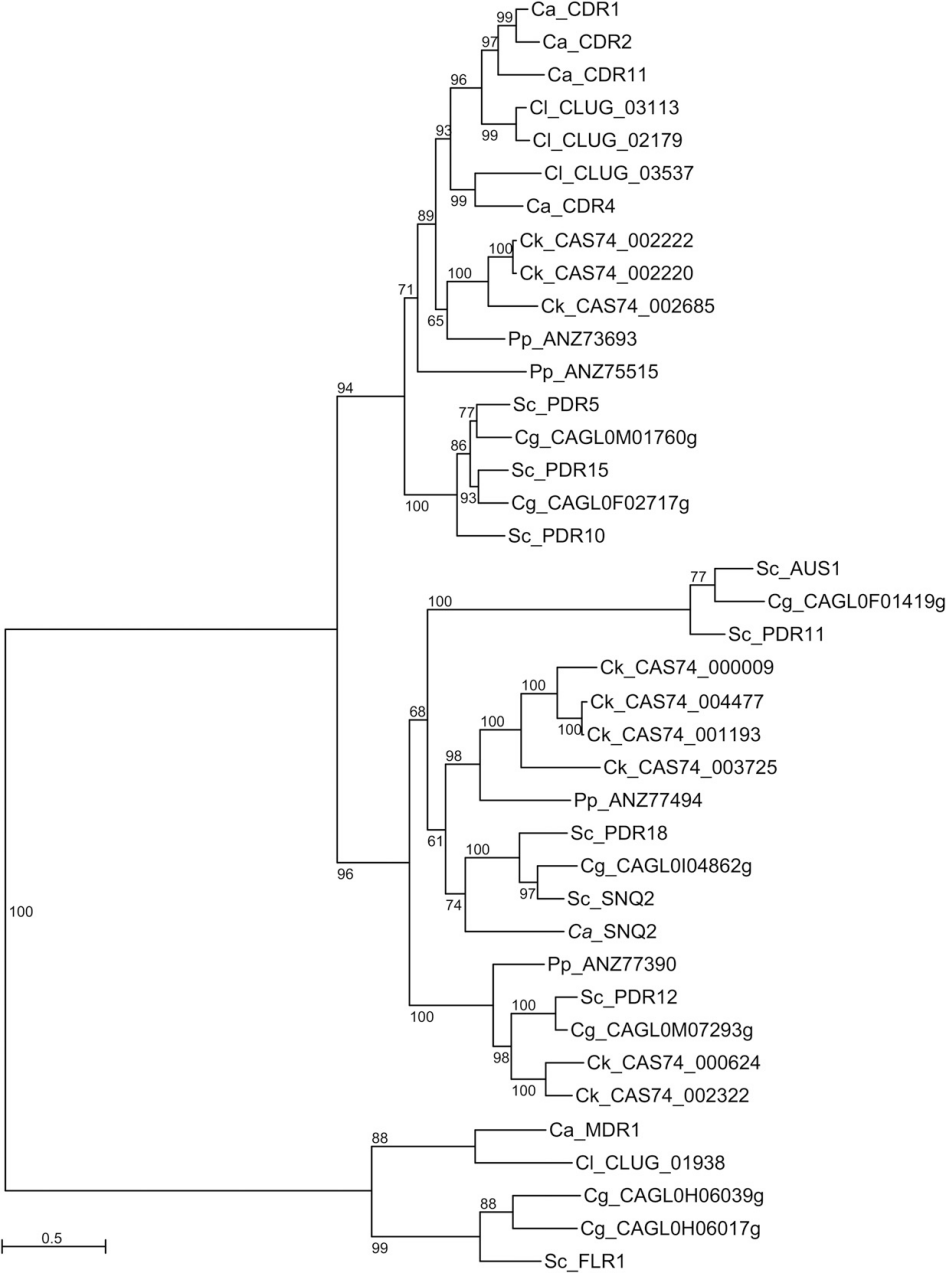
Phylogeny of Cdr and Mdr proteins in *C. krusei* and related species. Cdr and Mdr proteins identified across six species were aligned and used to infer a phylogeny using RAxML (*Materials and Methods*). Prefix for each protein corresponds to the species as follows: Ca, *C. albicans*; Cl, *C. lusitaniae*; Ck, *C. krusei*; Pp, *P. pastoris*; Cg, *C. glabrata*; Sc, *S. cerevisiae*.

To provide a preliminary view of the genes involved in pathogenesis and drug resistance, we identified orthologs of *C. albicans* genes in the *C. krusei* genome. Overall, gene families involved in pathogenesis in *C. albicans* are present in fewer copies in *C. krusei*. We identified fewer copies of the secreted aspartyl proteases, oligopeptide transporters, and phospholipase B genes (Table S2). In addition, we found no copies of genes similar to the secreted lipase or *ALS* cell surface families of proteins from *C. albicans*. This result is consistent with prior comparison to a wider set of pathogenic *Candida* more closely related to *C. albicans*, which observed expansion of several of these families in the more commonly pathogenic species (Butler *et al.* 2009). We also identified orthologs of genes noted to be involved in drug resistance in *C. albicans*, via point mutations, increased transcription, or copy number variation. *C. krusei* contains a single copy of the *ERG11* azole target and of each of the *TAC1* and *UPC2* transcription factors. Several of the sites often subject to drug resistant mutations in *C. albicans* are conserved in *C. krusei* (*i.e.*, Y132, K143, and F126), suggesting no intrinsic azole resistance due to mutation of these sites in *C. krusei*. While we did not identify a copy of the *MDR1* drug transporter, we identified nine candidate transporters related to *CDR1*, *CDR2*, and related genes (Figure 3). These include three *C. krusei* genes related to *CDR1*/*CDR2*/*CDR11*/*CDR4*, four genes related to *SNQ2*/*PDR18*, and two genes related to *PDR12*. This may suggest a very different capability for drug efflux.

**Figure 4:**
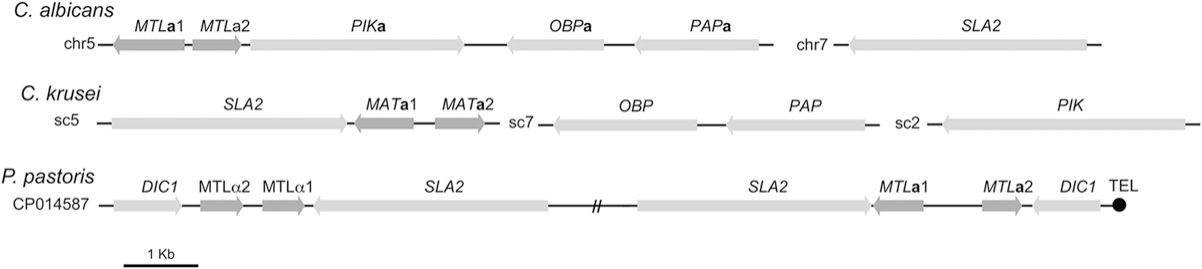
Mating type locus of *C. krusei*. Genes adjacent to the mating type locus of *C. krusei* differ from the CTG clade *Candida* and other related species; there is a single copy of *MAT***a**1 and *MAT***a**2 found in the assembly, adjacent to the *SLA2* gene, whereas the *OBP*, *PIK*, and *PAP* genes are found on other scaffolds in the assembly.

While previous genomic studies have revealed the highly variable content of the mating type locus in pathogenic *Candida* species (Butler *et al.* 2009), the mating type locus in *C. krusei* appears complete, and is more similar to that of Saccharomycetaceae yeasts than the CTG clade *Candida*. The mating type locus in *C. krusei* is found on scaffold 5, and includes the *MTL***a**1 gene and *MT*L**a**2 located adjacent to *SLA2* (Figure 4), similar to the configuration in many Saccharomycetaceae yeasts (Gordon *et al.* 2011). The mating type locus is close to the start of scaffold 7, separated from the end by four genes. Three other genes typically found at the mating locus of CTG clade *Candida* species (Butler *et al.* 2009) are located on adjacent scaffolds; *PAP1* and *OBPA* are adjacent on scaffold 7 and *PIKA* is on scaffold 2. While the related species *Pichia pastoris* and *Hansenula polymorpha* contain two *MAT* loci (Hanson *et al.* 2014), only one copy of *MTL***a**1, *MTL***a**2, and *SLA2* were found in the *C. krusei* assembly. This locus is potentially subtelomeric, as the start of the *SLA2* gene is 7.4 kb from the start of scaffold 5. The *MTL* region is heterozygous (Figure 1), as observed in some *MTL***a**/**a** and *MTL*α/α *C. albicans* isolates (Hirakawa *et al.* 2015). Both of the other assembled genomes of *C. krusei* also contain the *MTL***a** idiomorph, based on blastp to the available gene set for the 129 assembly or tblastn to the available assembly for M12. This information could guide a search for isolates of the opposite mating type, to begin to study whether *C. krusei* is capable of sexual reproduction.

## Supplementary Material

Supplemental material is available online at www.g3journal.org/lookup/suppl/doi:10.1534/g3.117.043547/-/DC1.
